# Endoscopic program with a scoring system for surveillance of metachronous esophageal cell carcinoma for older patients considering risk factors after endoscopic resection

**DOI:** 10.1007/s10388-024-01077-5

**Published:** 2024-08-09

**Authors:** Sakiko Naito, Masakatsu Fukuzawa, Hirokazu Shinohara, Yasuyuki Kagawa, Akira Madarame, Yohei Koyama, Hayato Yamaguchi, Yoshiya Yamauchi, Takao Itoi

**Affiliations:** Department of Gastroenterology and Hepatology, Tokyo Medical University, 6-7-1, Nishishinjuku, Shinjuku-ku, Tokyo, 167 0043 Japan

**Keywords:** Older patients, Endoscopic resection, Metachronous esophageal squamous cell carcinoma, Surveillance

## Abstract

**Background:**

This study evaluated the association between the risk factors and prognosis for metachronous esophageal squamous cell carcinoma (ESCC) after endoscopic resection (ER) of esophageal cancer in older patients.

**Methods:**

We conducted a retrospective observational study of 127 patients with ESCC who underwent ER from 2015 to 2020. Patients were classified as non-older (≤ 64 years), early older (65–74 years), and late older (≥ 75 years). We analyzed factors associated with poor overall survival and metachronous ESCC after ER using multivariate Cox regression analysis. A metachronous ESCC prediction scoring system was examined to validate the surveillance endoscopy program.

**Results:**

Body mass index (BMI) and Charlson Comorbidity Index (CCI) were significant risk factors for poor overall survival in the multivariate analysis (p = 0.050 and p = 0.037, respectively). Multivariate analysis revealed that age of < 64 years, Lugol-voiding lesions (grade B/C), and head and neck cancer were significantly related to metachronous ESCC (p = 0.035, p = 0.035, and p = 0.014, respectively). In the development cohort, BMI < 18.5 kg/m^2^, CCI > 2, age < 64 years, Lugol-voiding lesions (grade B/C), and head and neck cancer were significantly related to metachronous ESCC, and each case was assigned 1 point. Patients were classified into low (0, 1, and 2) and high (> 3) score groups based on total scores. According to Kaplan–Meier curves, the 3-year overall survival was significantly lower in the high-score group than in the low-score group (91.5% vs. 100%, p = 0.012).

**Conclusions:**

We proposed an endoscopic surveillance scoring system for metachronous ESCC considering BMI and CCI in older patients.

**Supplementary Information:**

The online version contains supplementary material available at 10.1007/s10388-024-01077-5.

## Introduction

Esophageal cancer is the eighth most common disease worldwide and has the sixth highest mortality rate. Further, this disease causes approximately 3.2% of all cancer-related deaths [1–4]. Endoscopic resection (ER) is a minimally invasive treatment method that allows for curative resection and can help preserve esophageal function [5]. Therefore, early diagnosis and prompt treatment are important for the management of this disease. In 2009, the World Health Organization (WHO) identified alcohol-associated acetaldehyde as a group 1 carcinogen [6]. Alcohol consumption is associated with the risk of damage to the esophageal mucosa. Specific risk factors include the amount of alcohol intake, alcohol consumption duration, and alcohol metabolism-related genotypes persisting in the esophageal mucosa [7–9]. Katada et al. [10] reported a relationship between alcohol consumption and the degree of esophageal mucosal damage in head and neck cancer (HNC) associated with field cancerization, as well as between esophageal mucosal damage grade and the risk of developing metachronous esophageal squamous cell carcinoma (ESCC) and HNC. With the advent of a super-aging society, medical care for older adults has become a major social issue. Frailty is associated with decreased quality of life and post-treatment effects among older adult patients, who are likely to have multiple comorbidities [11]. Therefore, this study aimed to evaluate the association between the risk factors and prognosis for metachronous ESCC after the endoscopic treatment of ESCC in older patients. Furthermore, this study examined the background factors associated with appropriate post-treatment care required to maintain the ability to perform activities of daily living and evaluated their associations with the prognosis.

## Methods

### Patients and study design

This single-center, retrospective, observational study enrolled 127 patients newly diagnosed with ESCC at Tokyo Medical University Hospital between 2015 and 2020. Patients were classified based on the WHO definition into the non-older (≤ 64 years), early-older (65–74 years), and late-older (≥ 75 years) groups [12]. The risk factors for overall survival (OS) were investigated using the American Society of Anesthesiologists (ASA)-Physical Status (PS) (ASA-PS) classification system [13] and the Charlson Comorbidity Index (CCI) [14, 15].

### LVL grades

Lugol-voiding lesion (LVL) grades A, B, and C were defined as no LVLs, 1–9 lesions, and ≥ 10 lesions identified with iodine staining, respectively [10].

### Follow-up endoscopic examinations post-ER

Patients with LVL grades B and C were expected to undergo endoscopy every 3–6 months while those with LVL grade A were expected to undergo endoscopy every year. The surveillance methods used included white light, narrow band imaging, and iodine staining for all cases following endoscopic treatment.

### Definition of synchronous and metachronous ESCC

Synchronous lesions were defined as lesions detected at the time of endoscopy in the endoscopically treated patients, while metachronous lesions were defined as new lesions detected 3 months after-surveillance endoscopy [10, 16].

### Statistical analysis

Quantitative data were compared using the Mann–Whitney U test. Categorical data were analyzed using Fisher’s exact test and the χ^2^ test. OS was defined as the time from ER until death or the last follow-up day. The survival time was measured from the date of the ER to the date of any death or the latest survival confirmation until January 31, 2023. Regarding the metachronous ESCC analysis, the conclusion of the follow-up period was determined to be the last endoscopy date until January 31, 2023. The duration until the metachronous ESCC occurrence was computed from the ER date to the time of detecting metachronous ESCC. Poor OS serves as a prognostic factor for OS. The cumulative incidence was determined as the cumulative morbidity of metachronous ESCC for the first event, and survival curves were calculated using the Kaplan–Meier method. The risk ratio and 95% confidence interval (CI) were estimated using the Cox proportional hazards model. Cox regression analysis was used in the development cohort to select prognostic factors for the scoring system predicting mortality post-ER. Specifically, multivariate analysis was performed for all the factors from the Cox hazard analysis, and those with p < 0.05 in the multivariable model were selected as prognostic factors for the scoring system. These statistical analyses were performed using SPSS software (version 27.0; IBM Japan, Tokyo, Japan). Statistical significance was set at p < 0.05. The optimal cut-off value for the scoring system was determined using Youden’s index, receiver operating characteristic (ROC) curve analysis, and the area under the curve (AUC). Time-dependent ROC analysis was conducted with the R package “time ROC” to handle censored survival data [17].

## Results

### Characteristics of patients and lesions

Overall, 127 patients were classified into the non-older (41 patients), early-older (46 patients), and late-older (40 patients) groups (Table 1). Metachronous ESCC occurrence was significantly higher in the non-older group than in the early-older and late-older groups (46.3%, 23.9%, and 25.0%, p = 0.045); they were also heavy drinkers (p = 0.004). The ASA-PS score (3–4) was significantly higher in the older (p = 0.013).

**Table 1:** Patients’ characteristics

Patients	< 64 years	65–74 years	> 75 years	p-value
	n = 41	n = 46	n = 40	
Age, median (IQR), years	60 (55–62)	69 (67–72.3)	78.5 (77–81)	< 0.001
Sex, male, n (%)	34 (82.3)	39 (84.8)	35 (87.5)	0.845
BMI, kg/m^2^, n (%)	20.8 (19.7–23.2)	22.3 (19.2–24.9)	21.7 (19.5–24.0)	0.261
Multiple ESCCs (1/2/3/4)	34/5/1/1	42/3/1/0	35/3/2/0	0.684
Metachronous ESCC, %	46.3	23.9	25.0	0.045
Metachronous ESCC (0/1/2/3/4/5)	22/14/4/0/1	35/9/2/0/0	30/8/1/1/0	0.040
Drinking alcohol (never/past/current)	5/6/30	8/4/34	10/7/23	0.197
Heavy alcohol consumption (never/sometimes/frequently)	6/8/27	6/18/22	10/9/21	0.318
Amount of alcohol intake (never/light/moderate/heavy)	5/5/7/24	6/19/6/15	10/14/6/10	0.004
Smoking (non-smoker/light/heavy), pack-years	14/14/13	7/15/24	11/10/19	0.082
Smoking (never/past/current)	15/25/1	7/37/2	11/29/0	0.059
Grade of LVLs (A/B/C)	2/8/31	4/9/33	4/10/26	0.544
Observation period, median (IQR), months	48.3 (33.7–69.5)	47.6 (35.0–58.4)	42.0 (27.9–59.0)	0.347
Metachronous period, median (IQR), months	33.6 (20.6–49.0)	43.8 (24.8–55.4)	33.9 (23.2–49.1)	0.500
ASA-PS, n (%)				
1–2	25 (61)	36 (78.3)	19 (47.5)	0.013
3–4	16 (39)	10 (21.7)	21 (52.5)	
CCI, n (%)				
0.1	28 (68.3)	21 (45.7)	28 (70)	0.871
> 2	13 (31.7)	14 (30.4)	12 (30)	
Double primary cancer, n (%)	8 (19.5)	11 (23.9)	10 (25)	0.823
Respiratory disfunction, n (%)	1 (2.4)	2 (4.3)	5 (12.5)	0.142
Heart disease, n (%)	0	1 (2.2)	5 (12.5)	0.018
Liver dysfunction, n (%)	1 (2.4)	0	0	0.355
Renal dysfunction, n (%)	0	0	1 (2.5)	0.377
Diabetes mellitus, n (%)	5 (12.1)	6 (13.0)	2 (5.0)	0.418
Antithrombotic internal rate, n (%)	2 (4.8)	1 (2.2)	12 (30)	< 0.001

Never: < 1 U/week; light: 1–9 U/week; moderate: > 9 to 18 U/week; heavy: > 18 U/week. U = 22 g of ethanol

*ASA-PS* American Society of Anesthesiologists–Performance Status, *BMI* body mass index, *CCI* Charlson Comorbidity Index, *ESCC* esophageal squamous cell carcinoma, *IQR* interquartile range, *LVL* Lugol-voiding lesion

### Treatment outcomes of ER

The en bloc resection rates of all groups were high (95.9%, 94.1%, and 98%, respectively), the median hospital stay was 7 days, and no differences were observed in adverse events for all groups. Surveillance endoscopy post-ER was performed every 6 months for all groups (Table 2).

**Table 2:** Treatment outcomes of ER

	< 64 years	65–74 years	> 75 years	p-value
	n = 49	n = 51	n = 50	
Lesions				
Size, median (IQR), mm	19 (12–27)	20 (11–28)	20 (11–28)	0.555
Location (Ce/Ut/Mt/Lt)	0/3/31/15	0/6/38/7	2/4/39/5	0.048
Macroscopic type (elevated/flat/depressed)	4/24/21	5/22/24	4/24/22	0.813
EMR/ESD	5/44	11/40	9/41	0.479
En bloc resection rate, %	95.9	94.1	98	0.794
Invasion depth, n (%)				
Pre-operative diagnosis depth, n (%)				0.409
EP	19 (38.8)	20 (39.2)	16 (32)	
LPM	26 (53.1)	23 (45.1)	24 (48)	
MM/SM1	4 (8.2)	8 (15.7)	10 (20)	
Pathological diagnosis depth, n (%)				0.367
EP	17 (34.7)	22 (43.1)	16 (32)	
LPM	24 (49.0)	20 (39.2)	21 (42)	
MM	5 (10.2)	6 (11.8)	9 (18)	
SM1	2 (4.1)	3 (5.9)	2 (4)	
SM2	1 (2.0)	0	2 (4)	
Lymphovascular invasion, n (%)	2 (4.1)	0	2 (4)	0.538
Vascular invasion, n (%)	2 (4.1)	1 (2.0)	5 (10)	0.325
Horizontal margin, n (%)	5 (10.2)	0	4 (8)	0.149
Vertical margin, n (%)	2 (4.1)	2 (3.9)	2 (4)	0.997

Patients	< 64 years	65–74 years	> 75 years	
	n = 41	n = 46	n = 40	
Adverse events, n (%)	4 (8.2)	3 (5.7)	3 (6.0)	0.852
Stenosis, n (%)	3 (7.3)	0	2 (5.0)	0.200
Perforation, n (%)	1 (2.4)	0	1 (2.5)	0.564
Pneumonia, n (%)	0	1 (2.2)	0	0.415
Mediastinal emphysema, n (%)	0	2 (3.3)	0	0.169
Length of hospital stay for ER, median (IQR), days	7 (7–8)	7 (7–9)	7 (7–8)	0.622
Endoscopic interval, median (IQR), months	6 (6–6)	6 (6–7)	6 (6–6)	0.210

*Ce* cervical esophagus, *EMR* endoscopic mucosal resection, *EP* epithelium, *ER* endoscopic resection, *ESD* endoscopic submucosal dissection, *IQR* interquartile range, *LPM* lamina propria muscle, *Lt* Lower thoracic esophagus, *MM* muscularis mucosae, *Mt* middle thoracic esophagus, *SM* submucosal layer, *Ut* upper thoracic esophagus

### Risk factors associated with poor 3-year survival for patients with ESCC who underwent ER

**Fig. 1:**
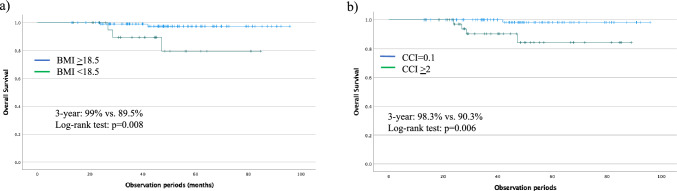
Overall survival (OS) rates of patients undergoing endoscopic resection (ER). **a** OS rates based on body mass index (BMI). **b** OS rates stratified according to the Charlson Comorbidity Index (CCI). BMI and CCI were significantly associated with OS

Body mass index (BMI) < 18.5 kg/m^2^ had a significant effect on the univariate analysis and was a significant factor in the multivariate analysis (p = 0.050). The 3-year survival rates were 99% and 89.5% according to the age groups with BMI > 18.5 and < 18.5 kg/m^2^, respectively (p = 0.008) (Fig. 1a). The receiver operating characteristic (ROC) curve analysis demonstrated that the area under the ROC curve was 0.757 (95% CI 0.547–0.966), and the optimal CCI cut-off value was 2. Additionally, the OS risk was investigated based on the CCI; 3-year survival rates of 98.3% and 90.3% were observed in patients with CCI 0.1 and > 2, respectively. CCI > 2 was associated with significantly lower OS rates (p = 0.006) (Fig. 1b). Multivariate analysis showed that BMI and CCI were significant risk factors for OS (p = 0.050 and p = 0.037, respectively) (Table 3). Five patients died after endoscopic treatment. Among them, 60% had a BMI < 18.5 kg/m^2^, 40% had ASA-PS scores of 3 or 4, and 60% had a CCI > 2. Furthermore, metachronous ESCC occurred in 40% of these patients. All deaths were caused by diseases other than ESCC, including heart disease (one patient), lung cancer (one patient), pleural mesothelioma (one patient), and HNC (two patients) (Supplementary Table).

**Table 3:** Risk factors associated with poor OS

	Univariate analysis			Multivariate analysis		
	HR	95% CI	p-value	HR	95% CI	p-value
OS						
Age, years						
Continuous variable	1.027	0.932–1.132	0.591	1.055	0.935–1.191	0.385
BMI, kg/m^2^						
< 18.5	7.685	1.282–46.071	0.026	8.956	0.990–80.990	0.050
> 18.5	1.0	Reference		1.0	Reference	
ASA-PS						
1–2	1.0	Reference		1.0	Reference	
3–4	2.695	0.448–16.196	0.279	4.600	0.598–35.413	0.143
CCI						
0.1	1.0	Reference		1.0	Reference	
> 2	11.458	1.278–102.748	0.029	13.460	1.171–154.751	0.037
Curative resection						
Noncurative	2.508	0.277–22.729	0.414	0.527	0.035–7.844	0.642
Curative	1.0	Reference		1.0		

*ASA-PS* American Society of Anesthesiologists-Performance Status, *BMI* body mass index, *CCI* Charlson Comorbidity Index, *OS* overall survival

### Risk factors associated with the cumulative incidence of metachronous ESCC

**Fig. 2:**
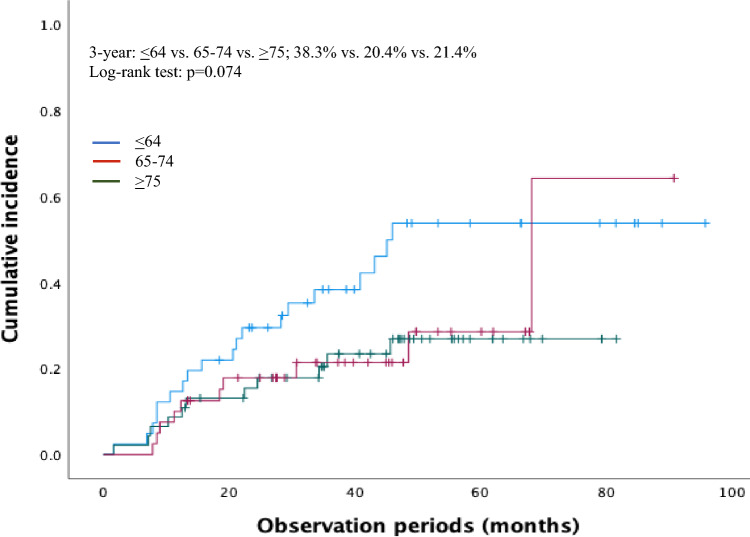
Cumulative incidence rate of patients undergoing endoscopic resection**.** Cumulative incidence based on age (≤ 64 years vs. 65–74 years vs. ≥ 75 years). No difference was found in the 3-year cumulative incidence rates based on age

Metachronous ESCC incidence in the non-older, early-older, and late-older groups were 46.3%, 23.9%, and 25.0%, respectively; this was significantly higher among non-older patients (p = 0.045). However, no difference was found in the 3-year cumulative incidence rates based on age (38.3% vs. 20.4% vs. 21.4%, p = 0.074) (Table 4, Fig. 2). Metachronous ESCC occurred at a median of 33.6, 43.8, and 33.9 months in the non-older, early-older, and late-older groups, respectively (Table 1). During the multivariate analysis, non-older age, LVLs, and HNC prevalence were significant (p = 0.035, p = 0.035, and p = 0.014, respectively).

**Table 4:** Risk factors associated with the cumulative incidence of metachronous ESCC

	Univariate			Multivariate		
	HR	95% CI	p*-*value	HR	95% CI	p-value
Cumulative incidence						
Age, years						
Continuous variable	0.957	0.924–0.991	0.014	0.961	0.925–0.997	0.035
Sex						
Male	1.242	0.548–2.813	0.603	2.134	0.854–5.333	0.105
Female	1.0	Reference		1.0	Reference	
BMI, kg/m^2^						
< 18.5	1.276	0.607–2.683	0.521	0.675	0.284–1.603	0.373
> 18.5	1.0	Reference		1.0	Reference	
LVLs						
Grade A	1.0	Reference		1.0	Reference	
Grade B/C	2.181	1.070–4.444	0.032	2.252	1.059–4.786	0.035
Heavy alcohol consumption						
Never/sometimes	1.0	Reference		1.0	Reference	
Frequently	1.886	0.984–3.617	0.056	1.125	0.370–3.419	0.835
Amount of alcohol intake (units/week)						
Never/light	1.0	Reference		1.0	Reference	
Moderate/heavy	2.084	1.073–4.046	0.030	1.498	0.466–4.817	0.498
Smoking, pack-years						
Nonsmoker/light, < 30	1.0	Reference				
Heavy, > 30	1.133	0.607–2.117	0.694	1.144	0.594–2.205	0.687
Multiple ESCCs (–)	1.0	Reference		1.0	Reference	
Multiple ESCCs (+)	1.237	0.518–2.957	0.632	0.853	0.33–2.183	0.740
Head and neck						
Never	1.0	Reference		1.0	Reference	
Past/current	2.629	1.399–4.939	0.003	1.498	1.193–4.845	0.014
Lung carcinoma						
Never	1.0	Reference				
Past/current	0.045	0.000–33.966	0.360		–	

*BMI* body mass index, *ESCC* esophageal cell carcinoma, *LVL* Lugol-voiding lesion

A summary of the causes of death after ER is provided in Supplementary table. Five patients died after endoscopic treatment. The median age of the patients who died was 70 years (interquartile range [IQR], 69–71 years), and all patients were male. Among them, 60% had a BMI < 18.5 kg/m^2^, 40% had ASA-PS scores of 3 or 4, and 60% had a CCI > 2. Furthermore, metachronous ESCC occurred in 40% of these patients. All deaths were caused by diseases other than ESCC, including heart disease (one patient), lung cancer (one patient), pleural mesothelioma (one patient), and HNC (two patients).

### Development of a prognostic scoring system of metachronous ESCC

**Fig. 3:**
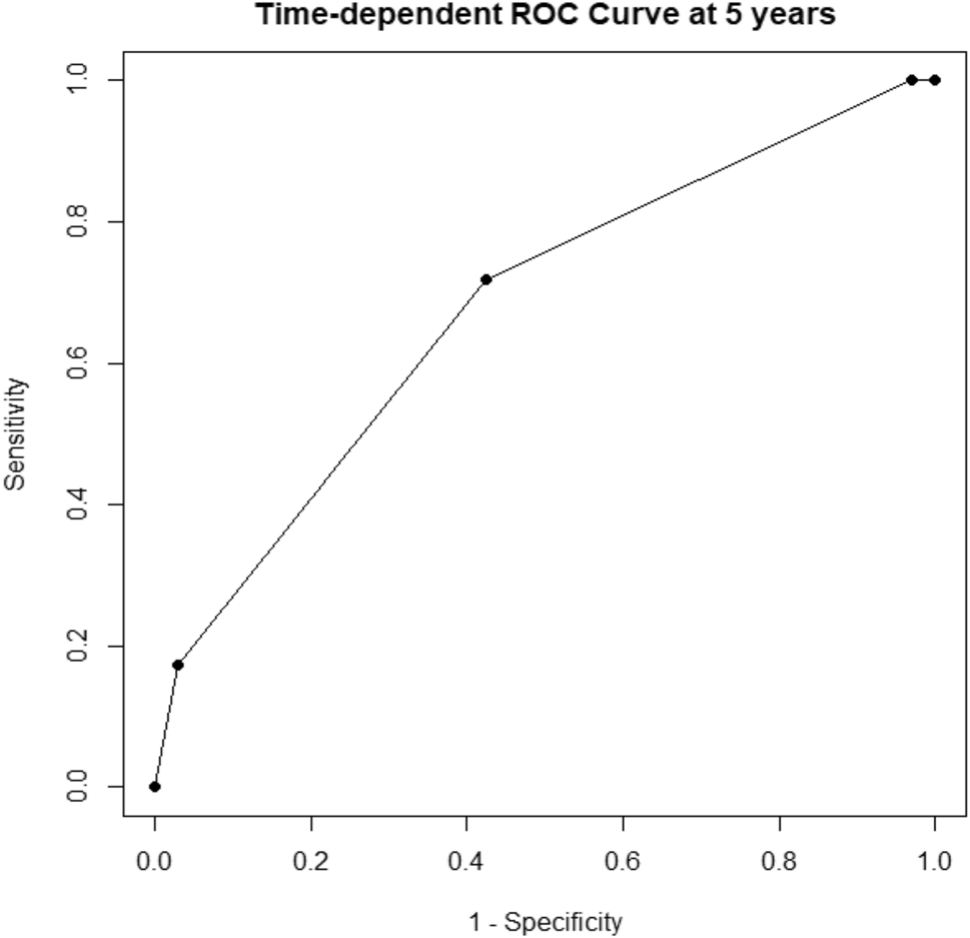
Receiver operating characteristic curve for the scoring system: time-dependent receiver operating characteristic (ROC) curve at 5 years for overall survival (OS). The area under the curve was 0.677, with an optimal cut-off value of 2

**Fig. 4:**
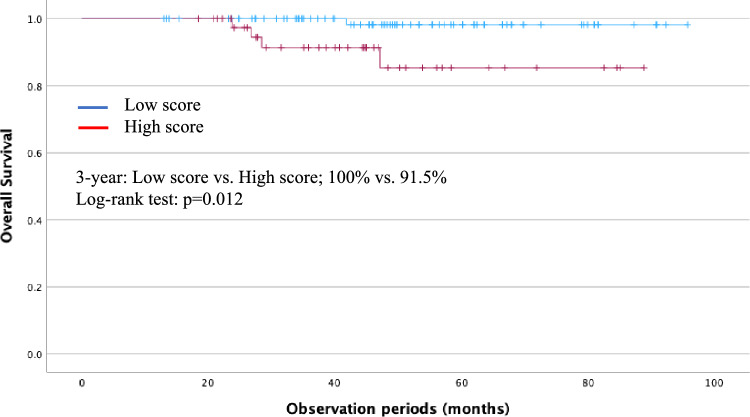
Development of a prognostic scoring system for metachronous esophageal squamous cell carcinoma (ESCC). Comparison of OS rates between the low- and high-score groups of patients in the validation cohort. The 3-year OS incidence rates were significantly lower in the high-score group than in the low-score group (91.5% vs. 100%, p = 0.012)

Cox analysis of risk factors for poor OS after-ER showed that BMI < 18.5 kg/m^2^ and CCI > 2, respectively, were significant factors in the multivariate analysis. Cox analysis of risk factors for metachronous ESCC after-ER showed that less than 64, LVLs (grade B/C), and history of HNC were significant factors in multivariate analysis. We assigned points proportional to the regression coefficient for each of the three predictive variables to calculate the risk score: 1 point each for BMI < 18.5 kg/m^2^, CCI > 2, 1 point; < 64, LVLs (B/C), and history of HNC. Therefore, the maximum score was 5 points. Using the scoring system, scores of 0, 1, 2, 3, 4, and 5 points were assigned to 2, 44, 38, 30, 10, and 3 patients in the development cohort, respectively. Time-dependent ROC analysis of OS was conducted, yielding an AUC of 0.677 and a Youden’s Index of 0.294, with a cut-off value of 2 (Fig. 3). Patients with scores of 0–2 and 3–5 points were categorized as the low- and high-score groups, respectively. The low- and high-score groups comprised 84 and 43 patients, respectively. The median recurrence period was 40 and 28 months in the low-score and high-score groups, respectively (p = 0.011) (Table 5), indicating a significantly shorter period of metachronous ESCC (p = 0.011). The 3-year OS was significantly lower in the high-score group than in the low-score group (91.5% vs. 100%, p = 0.012) (Fig. 4).

**Table 5:** Characteristics of each of the score groups

Patients	Low score	High score	p-value
	n = 84	n = 43	
Age, median (IQR), years	72 (65–77)	67 (60–72)	0.011
Sex, male, n (%)	74 (88.1)	34 (79.1)	0.196
Metachronous ESCC period, median (IQR), months	40 (26–56)	28 (19–44)	0.011

*IQR* interquartile range, *ESCC* esophageal cell carcinoma

## Discussion

A consensus has not been reached on the optimal surveillance endoscopy following treatment for superficial ESCC. Moreover, frequent uncertainty exists concerning the prognosis after treatment by surveillance. Therefore, we evaluated scorings, including BMI and CCI, which are significant risk factors for OS and stratified the risk contributing to OS in metachronous ESCC after endoscopic treatment.

ER is a minimally invasive procedure that can be safely performed [5, 18]. However, older individuals have high rates of comorbidities; therefore, the impact of comorbidities on treatment and factors contributing to the prognosis should be considered. The CCI is useful for predicting the prognosis of older patients [14, 19, 20]. In our study, the 3-year OS rates were 99% and 89.5% in non-older and older patients with lower BMI, respectively. Patients with CCI > 2 had significantly poorer OS rates; the 3-year OS rates were 98.3% and 90.3% for patients with CCI = 0.1 and CCI > 2, respectively. According to the univariate and multivariate analyses, BMI and CCI were significant risk factors for an adverse prognosis. BMI is positively associated with body fat percentage in patients with low skeletal muscle and plays a central role in immune function. Therefore, there may be a central predictor of prognosis in patients with esophageal cancer [21]. Different mechanisms have shown that both low nutrition and high weight are associated with poor prognosis in ESCC, with advanced tumor and tumor biologic invasiveness being the causes of poor prognosis in patients with low and high BMI, respectively [22]. However, no reports of BMI associated with metachronous ESCC post-ER exist. This may serve as a reference for future therapeutic targets for endoscopic treatment. Here, the higher number of older patients with ASA-PS scores of 3–4 suggests that thorough evaluation of the general health condition should be an essential component of the pre-operative assessment when minimally invasive endoscopic treatment is an option. Therefore, surveillance that considers BMI, CCI, and ASA-PS may be warranted. This approach underscores the potential impact of suitable surveillance on prognosis, highlighting the importance of incorporating screening for risk within such a surveillance program. Surveillance endoscopy is essential for the early diagnosis of metachronous ESCC after endoscopic treatment. Additionally, follow-up endoscopy and other imaging studies are important post-ER because ESCC is associated with the development of metachronous ESCC and secondary cancers post-ER. In this study, the cumulative incidence of metachronous ESCC was higher among non-older patients than among older patients; grade (B/C) LVLs and HNC were lower among older patients than among non-older patients, according to the Cox hazard analysis. Non-older patients with ESCC may be at higher risk of metachronous ESCC and HNC because of their greater exposure to risk factors, such as alcohol consumption and tobacco use. The incidence rate of metachronous ESCC was higher among non-older patients with HNC comorbidity than among older patients [23], and the incidence rate of secondary cancers among non-older patients was higher because of their greater exposure to alcohol consumption and carcinogens [24]. More non-older patients were heavy drinkers; however, many older patients were non-drinkers and past drinkers, which could have affected metachronous ESCC prevalence. Metachronous ESCC and HNC are associated with field cancerization [9, 10], with HNC incidence rates of 5.1–7.3% post-ER for ESCC [25]. Of the five patients who died, four (80%; two with lung cancer and two with HNC) were older. No deaths were caused by metachronous ESCC; therefore, it is important to carefully evaluate the prognostic factors associated with esophageal cancer and perform appropriate imaging evaluations to search for secondary cancers. Alongside frequent surveillance endoscopy for ESCC in patients with multiple LVLs, HNC, and lower BMI, screening may detect the early diagnosis of the disease [26]. Therefore, risk factors, including those affecting OS, such as BMI and CCI, should be systematically stratified for metachronous ESCC.

This study had some limitations. First, it was a single-institution, retrospective study with a small number of patients. Second, the effect of appropriate endoscopic treatment on the survival of older patients with metachronous ESCC may have been attributed to the availability of proper endoscopic treatment. Third, we acknowledge a limitation regarding the variation in the surveillance endoscopy intervals among attending physicians, which could potentially impact the detection rate. Fourth, information regarding the risk factors for metachronous ESCC is limited because the results were obtained from a small number of participants; therefore, a multi-institutional study should be conducted in the future. Finally, frequent surveillance endoscopy may not have affected the OS of older patients with low BMI or high CCI; therefore, another follow-up interval may be acceptable.

In conclusion, we proposed an endoscopic surveillance scoring system for metachronous ESCC, considering BMI and CCI in older patients.

## Supplementary Information

Below is the link to the electronic supplementary material.

Supplementary file1 (DOCX 15 KB)

## Data Availability

The datasets generated and/or analyzed in this study are not publicly available but are available from the corresponding author upon reasonable request.

## References

[1] Kitagawa Y, Uno T, Oyama T, et al. Esophageal cancer practice guidelines 2017 edited by the Japan Esophageal Society: Part 1. Esophagus. 2019;16:1–24. 30171413 10.1007/s10388-018-0641-9 PMC6510883

[2] Kitagawa Y, Uno T, Oyama T, et al. Esophageal cancer practice guidelines 2017 edited by the Japan Esophageal Society: Part 2. Esophagus. 2019;16:25–43. 30171414 10.1007/s10388-018-0642-8 PMC6510875

[3] Pennathur A, Gibson MK, Jobe BA, et al. Oesophageal carcinoma. Lancet. 2013;381:400–12. 23374478 10.1016/S0140-6736(12)60643-6

[4] Ferlay J, Soerjomataram I, Dikshit R, et al. Cancer incidence and mortality worldwide: sources, methods and major patterns in GLOBOCAN 2012. Int J Cancer. 2015;136:E359–86. 25220842 10.1002/ijc.29210

[5] Ishihara R, Iishi H, Uedo N, et al. Comparison of EMR and endoscopic submucosal dissection for en bloc resection of early esophageal cancers in Japan. Gastrointest Endosc. 2008;68:1066–72. 18620345 10.1016/j.gie.2008.03.1114

[6] Secretan B, Straif K, Baan R, et al. A review of human carcinogens-part E: tobacco, areca nut, alcohol, coal smoke, and salted fish. Lancet Oncol. 2009;10:1033–4. 19891056 10.1016/s1470-2045(09)70326-2

[7] Yokoyama A, Tsutsumi E, Imazeki H, et al. Salivary acetaldehyde concentration according to alcoholic beverage consumed and aldehyde dehydrogenase-2 genotype. Alcohol Clin Exp Res. 2008;32:1607–14. 18616675 10.1111/j.1530-0277.2008.00739.x

[8] Muto M, Nakane M, Hitomi Y, et al. Association between aldehyde dehydrogenase gene polymorphisms and the phenomenon of field cancerization in patients with head and neck cancer. Carcinogenesis. 2002;23:1759–65. 12376487 10.1093/carcin/23.10.1759

[9] Slaughter DP, Southwick HW, Smejkal W. Field cancerization in oral stratified squamous epithelium; clinical implications of multicentric origin. Cancer. 1953;6:963–8. 13094644 10.1002/1097-0142(195309)6:5<963::aid-cncr2820060515>3.0.co;2-q

[10] Katada C, Yokoyama T, Yano T, et al. Alcohol consumption and multiple dysplastic lesions increase risk of squamous cell carcinoma in the esophagus, head, and neck. Gastroenterology. 2016;151:860-69.e7. 27492616 10.1053/j.gastro.2016.07.040

[11] Tanaka T, Suda K, Ueno M, et al. Impact of frailty on the long-term outcomes of elderly patients with esophageal squamous cell carcinoma. Gen Thorac Cardiovasc Surg. 2022;70:575–83. 35334065 10.1007/s11748-022-01807-5

[12] World Health Organisation. Definition of an older or elderly person. Geneva: Switzerland; 2010. http://www.who.int/healthinfo/survey/ageingdefnolder/en/index.html. Accessed 12 Nov 2013.

[13] American Society of Anesthesiologists Committee on Economics. ASA physical status classification system, 2020. https://ASA-PShq.org/resources/clinical-information/ASA-PS-physical-status-classification-system. Accessed 13 Dec 2020.

[14] Charlson ME, Pompei P, Ales KL, et al. A new method of classifying prognostic comorbidity in longitudinal studies: development and validation. J Chronic Dis. 1987;40:373–83. 3558716 10.1016/0021-9681(87)90171-8

[15] Charlson M, Szatrowski TP, Peterson J, et al. Validation of a combined comorbidity index. J Clin Epidemiol. 1994;47:1245–51. 7722560 10.1016/0895-4356(94)90129-5

[16] SEER. Program code manual. 3rd ed. Bethesda: National Cancer Institute; 1998.

[17] Blanche P, Dartigues JF, Jacqmin-gadda H. Estimating and comparing time-dependent areas under receiver operating characteristics curves for censored event times with competing risks. Stat Med. 2013;32:5381–97. 24027076 10.1002/sim.5958

[18] Kuwano H, Nishimura Y, Oyama T, et al. Guidelines for diagnosis and treatment of carcinoma of the esophagus April 2012 edited by the Japan Esophageal Society. Esophagus. 2015;12:1–30. 25620903 10.1007/s10388-014-0465-1 PMC4297610

[19] Tominaga T, Nonaka T, Takeshita H, et al. The Charlson comorbidity index as an independent prognostic factor in older colorectal cancer patients. Indian J Surg. 2018;80:54–60. 29581686 10.1007/s12262-016-1544-4 PMC5866797

[20] Nakajo K, Abe S, Oda I, et al. Impact of the Charlson comorbidity index on the treatment strategy and survival in elderly patients after non-curative endoscopic submucosal dissection for esophageal squamous cell carcinoma: a multicenter retrospective study. J Gastroenterol. 2019;54:871–80. 31055660 10.1007/s00535-019-01583-9

[21] Kim GW, Nam JS, Abidin M, et al. Impact of body mass index and sarcopenia on short- and long- term outcomes after esophageal cancer surgery: an observational study. Ann Surg Oncol. 2022;29:6871–81. 35622181 10.1245/s10434-022-11944-z

[22] Watanabe M, Ishimoto T, Baba Y, et al. Prognostic impact of body mass index in patients with squamous cell carcinoma of the esophagus. Ann Surg Oncol. 2013;20:3984–91. 23797753 10.1245/s10434-013-3073-8

[23] Maekawa A, Ishihara R, Iwatsubo T, et al. High incidence of head and neck cancers after endoscopic resection for esophageal cancer in younger patients. J Gastroenterol. 2020;55:401–7. 31813008 10.1007/s00535-019-01653-y

[24] Iwatsubo T, Ishihara R, Morishima T, et al. Impact of age at diagnosis of head and neck cancer on incidence of metachronous cancer. BMC Cancer. 2019;19:3. 30606157 10.1186/s12885-018-5231-7 PMC6318848

[25] Shimizu Y, Tsukagoshi H, Fujita M, et al. Head and neck cancer arising after endoscopic mucosal resection for squamous cell carcinoma of the esophagus. Endoscopy. 2003;35:322–6. 12664389 10.1055/s-2003-38151

[26] Van de Ven S, Bugter O, Hardillo JA, et al. Screening for head and neck second primary tumors in patients with esophageal squamous cell cancer: a systematic review and meta-analysis. United Eur Gastroenterol J. 2019;7:1304–11. 10.1177/2050640619856459 PMC6893998 31839955

