# Supplementary material for: Endoscopic program with a scoring system for surveillance of metachronous esophageal cell carcinoma for older patients considering risk factors after endoscopic resection
Source: Esophagus. 2024 Aug 9;21(4):530–8. doi: 10.1007/s10388-024-01077-5 (PMC11405441; doi:10.1007/s10388-024-01077-5)
Supplement: Supplementary file 1 — Supplementary file1 (DOCX 15 KB) [file 10388_2024_1077_MOESM1_ESM.docx]

| **Supplementary Table.** Causes of death among patients treated with ER | | | | |  |  |  |
| --- | --- | --- | --- | --- | --- | --- | --- |
| Patient | Age, y | Sex | BMI, kg/m^2^ | ASA-PS (score) | CCI  (score) | Metachronous ESCC | Cause of death |
| 1 | 92 | Male | 19.3 | 3 | 1 | 1 | Heart disease |
| 2 | 70 | Male | 11.7 | 1 | 1 | 0 | Lung carcinoma |
| 3 | 71 | Male | 15.1 | 1 | 2 | 0 | Lung tumor |
| 4 | 69 | Male | 21.4 | 1 | 3 | 1 | HNC |
| 5 | 52 | Male | 16.2 | 3 | 2 | 0 | HNC |

Abbreviations: ASA-PS, American Society of Anesthesiologists–Performance Status; BMI, body mass index; CCI, Charlson Comorbidity Index; ER, endoscopic resection; ESCC, esophageal squamous cell carcinoma; HNC, head and neck cancer.
